# Hydroceles of the Canal of Nuck in Adults—Diagnostic, Treatment and Results of a Rare Condition in Females

**DOI:** 10.3390/jcm9124026

**Published:** 2020-12-12

**Authors:** Panagiotis Fikatas, Ioannis-Fivos Megas, Kiriaki Mantouvalou, Ibrahim Alkatout, Sascha S. Chopra, Matthias Biebl, Johann Pratschke, Jonas Raakow

**Affiliations:** 1 Department of Surgery, Campus Charité Mitte and Campus Virchow-Klinikum, Charité—Universitätsmedizin, Corporate Member of Freie Universität Berlin, Humboldt-Universität zu Berlin, and Berlin Institute of Health, Augistenburger Platz 1, 13353 Berlin, Germany; fivos.megas@gmail.com (I.-F.M.); kiriaki.mantouvalou@charite.de (K.M.); sascha.chopra@charite.de (S.S.C.); matthias.biebl@charite.de (M.B.); johann.pratschke@charite.de (J.P.); jonas.raakow@charite.de (J.R.); 2 Department of Obstetrics and Gynecology, Kiel School of Gynecological Endoscopy, University Hospital Schleswig Holstein, Campus Kiel, Arnold-Heller-Straße 3, 24105 Kiel, Germany; Ibrahim.Alkatout@uksh.de

**Keywords:** cysts of the canal of Nuck, Nuck hydrocele, hydrocelectomy, TAPP, Lichtenstein

## Abstract

Nuck’s hydroceles, which develop in a protruding part of the parietal peritoneum into the female inguinal canal, are rare abnormalities and a cause of inguinal swelling, mostly resulting in pain. They appear when this evagination of the parietal peritoneum into the inguinal canal fails to obliterate. Our review of the literature on this topic included several case reports and two case series that presented cases of Nuck hydroceles which underwent surgical therapy. We present six consecutive cases of symptomatic hydroceles of Nuck’s canal from September 2016 to January 2020 at the Department of Surgery of Charité Berlin. Several of these patients had a long history of pain and consecutive consultations to outpatient clinics without diagnosis. These patients underwent laparoscopic or conventional excision and if needed simultaneous hernioplasty in our institution. Ultrasonography and/or Magnetic Resonance Imaging were used to display the cystic lesion in the inguinal area, providing the diagnosis of Nuck’s hydrocele. This finding was confirmed intraoperatively and by histopathological review. Ultrasound and magnetic resonance imaging (MRI) captures, intraoperative pictures and video of minimal invasive treatment are provided. Nuck’s hydroceles should be included in the differential diagnosis of an inguinal swelling. We recommend an open approach to external Type 1 Nuck´s hydroceles and a laparoscopic approach to intra-abdominal Type 2 Nuck hydroceles. Complex hydroceles like Type 3 have to be evaluated individually, as they are challenging and the surgical outcome is dependent on the surgeon’s skills. If inguinal channel has been widened by the presence of a Nuck’s hydrocele, a mesh plasty, as performed in hernia surgery, should be considered.

## 1. Introduction

The canal of Nuck was first described by the Dutch anatomist Anton Nuck in 1691. As the female fetus develops, the ligamentum rotundum of the uterus descends down to the ipsilateral labia majora, extending through the inguinal canal. Along with the round ligament, a peritoneal evagination also descends, which is known as the canal of Nuck. The homologous structure in men is called the processus vaginalis [1].

More precise embryologically, the processus vaginalis—in women named canal of Nuck—becomes clinically apparent within the 12th week of gestation. Normally it obliterates from the seventh month of gestation to one year of age. Persistently open canals of Nuck present most often in girls before the age of five [1,2,3,4]. The Nuck hydrocele corresponds to the male hydrocele testis [4]. Nuck’s hydroceles or inguinal hernias occur in 9–11% of infants born prematurely, as the obliteration of the processus vaginalis begins during pregnancy [5].

Classification divides the Nuck hydroceles into three types [6]:

- Type 1: there is no communication between hydrocele and peritoneal cavity. It mostly appears as an encysted mass without hernia defect in children. Examples for this type are the intra-abdominal protruding forms. In adults, we assume the fascia transversalis along with the ligamentum rotundum is thinned out because of the hydrocele, mimicking a direct hernia [6].
- Type 2: the hydrocele communicates with the peritoneal cavity, thus mostly resulting in an indirect hernia [6].
- Type 3: or combined type has an encysted part that does not communicate with the peritoneal cavity and another that does. Its appearance resembles an hourglass and commonly causes a hernia [6].

To have a successful clinical outcome after surgery, complete excision of the hydrocele is recommended [7]. Following that, if a hernia defect is identified it requires repair by hernioplasty. There are about 134 publications on PubMed covering this topic, but only few case series and even fewer that compare the different surgical therapy options with each other. Especially, hydrocele of the canal of Nuck in adults have been reported only in single case presentations. To our knowledge, no case series with a cohort of adult females with Nuck’s hydrocele has been published so far. 

In our case series, we present six patients aged 29 to 44 with hydrocele of the canal of Nuck. Four of them received a Transabdominal Preperitoneal Patch Plasty (TAPP), one had an open hernia repair using the Lichtenstein method and another one an open hydrocelectomy along with a fascial suture, because there was no defect of the abdominal wall. We aim to share our experience on this rare condition and demonstrate that both types of hernioplasty can be performed for repairing a hernia caused by a Nuck hydrocele according to localization. Furthermore, we reviewed the case reports and case series published so far and compared their results and conclusions.

## 2. Methods

From 2016 to 2020, six cases of Nuck’s hydroceles presented to our Department of Surgery at Charité Universitätsmedizin Berlin. We retrospectively analyzed the collected data in all of these cases with regard to patient demographics, presenting symptoms, diagnostic workup, operative procedures and postoperative course.

The literature search was conducted with PubMed and Google Scholar using the following keywords: “nuck”, “nuck’s hydrocele”, “surgery”, “nuck hernia” and “canal of nuck”. In addition, we cross-checked reference lists from eligible publications and relevant review articles to identify additional studies. The inclusion criteria contained case reports or case series, with the main diagnosis of a “nuck’s hydrocele” or “nuck cyst” and included a surgical therapy. Exclusion criteria for case reports was the absence of any surgical therapy.

Surgical procedure: both conventional and laparoscopic approaches were used for exploration. 

Laparoscopic hydrocelectomy and TAPP:

Open access methods were used to place a laparoscopic trocar into the umbilicus for carbon dioxide (CO_2_) pneumoperitoneum. The peritoneum was opened above the spina iliaca anterior superior which then revealed a hydrocele of the canal of Nuck and an indirect hernia. The hydroceles were excised laparoscopically and a TAPP with a 10 × 15 cm polypropylene mesh placed over the hernia and glued with 1ml of fibrin before suturing the peritoneal flap.

Open hydrocelectomy and Lichtenstein:

After skin incision the external oblique aponeurosis is opened in the direction of the fibers. Preparation of the structures up to the cyst roof. Exposure of the Nuck’s hydrocele. Excision and high ligation of the hydrocele. Restoration of the anatomical structures and placement of a 12 × 6 cm polypropylene mesh. If the abdominal wall is intact, only a fascial suture was performed. Layer-appropriate closure and insertion of a drainage.

## 3. Results/Case Series Presentation

### 3.1. Case 1

A 29-year-old female presented in September 2016 with right-sided painful swelling in her inguinal region for one week. Both pain and size were increasing since she first noticed the swelling. There was no fever, no vomiting, no bowel or bladder dysfunction. On examination the swelling was painful and a manual reduction was not possible. There was no peristaltic activity or signs of inflammation. 

Ultrasonography showed a well-defined, 3 × 2 cm fluid-filled mass with discreet increase in size during Valsalva maneuver and there was a viewable connection to the inguinal canal.

The patient decided to proceed with elective exploration, hydrocelectomy of the type 2 and TAPP hernioplasty. The early postoperative period was uneventful and the patient was discharged home two days after surgery in satisfactory condition. On routine follow up, an inguinal seroma of 3.9 × 0.8 cm occurred three weeks postoperatively which was self-absorbed as it had disappeared in the subsequent follow-up under conservative therapy. The patient has remained asymptomatic on follow up lasting 6 months.

### 3.2. Case 2

**Figure 1:**
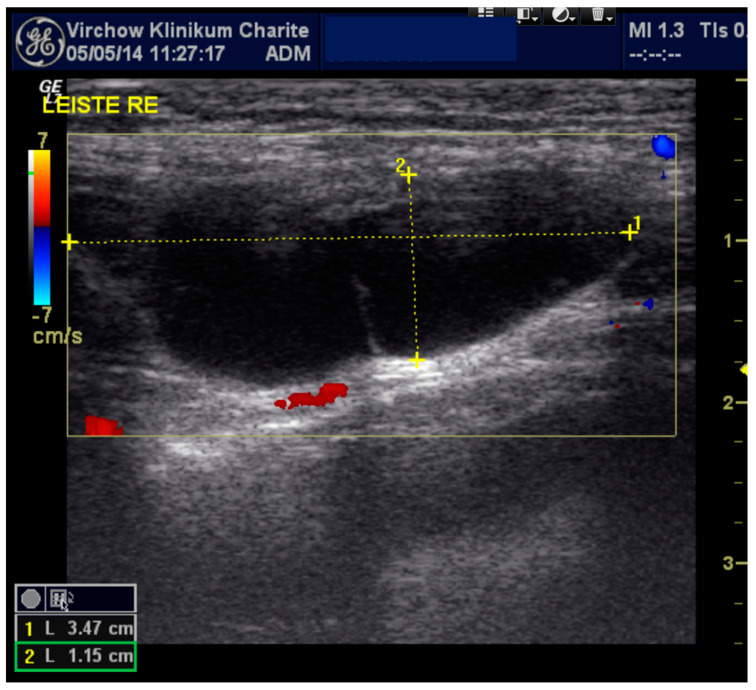
Sonographic imaging of the right inguinal area: anechoic elongated fluid structure 3.47 × 1.15 cm.

**Figure 2:**
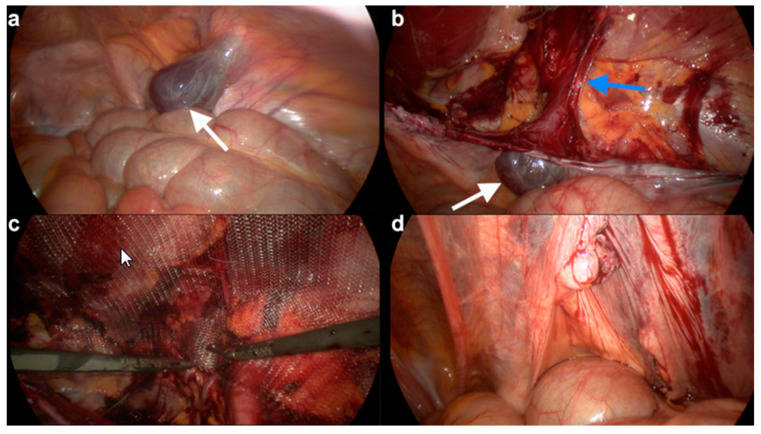
Laparoscopic procedures for hydrocelectomy and TAPP. (**a**) In the laparoscopic view, a type 2 hydrocele of the canal of Nuck (white arrow) (**b**) Approach to the inguinal canal and dissection of the hydrocele (white arrow) attached to the round ligament (below blue arrow). (**c**) Placement of the polypropylene mesh. (**d**) Sutured peritoneum.

A 29-year-old female presented to a gynecological ambulance in 2014 with an unclear right-sided inguinal mass. She first noticed it because of pain after doing sports and visited a hospital. A small seroma measuring 3.47 × 1.15 cm in the right groin region extending towards the vulva was found by ultrasound (Figure 1). At that time, it had not been deemed as requiring puncture. Two years later, in June 2016, she noticed that the swelling was growing in size and presented again. The magnetic resonance imaging (MRI) finding in T2 weighted imaging of a 11.1 × 3.4 cm hyperintense mass without wall-enhancement of the contrast agent (Gadovist) now resembled a cyst rather than a seroma. It was punctured and 40 mL of serous liquid was aspirated. The results of the examination revealed mesothelial cells and lymphocytes matching the findings of a lymphocele with a connection to the abdominal cavity. Taking into account the location of the cyst the diagnosis of a Nuck’s hydrocele was made. The patient was referred to our clinic five months later, in November 2016, and underwent exploration, hydrocelectomy of the type 2 and TAPP (Figure 2 and Video S1) hernioplasty.

The histopathological examination showed a mesothelium-coated cyst wall with chronic macrophage-rich inflammation and was consistent with those of a Nuck hydrocele. The patient was discharged two days after surgery and her follow up remains uneventful 6 months postoperatively.

The demonstrated image and video material in Figure 1 (preoperative sonography) and Figure 2 (intraoperative laparoscopic images and video) originates from this case.

### 3.3. Case 3

A 44-year-old female with multiple sclerosis was referred by her general practitioner to our general surgery department in July 2016 with a known cystic structure in the left inguinal region. The swelling was painful to touch and had increased in size over the last 2 months. Ultrasonography revealed a 3 × 4 cm liquid mass, with non-echoic content and without septations.

We performed a laparoscopic excision of the type 2 hydrocele and a left sided inguinal hernioplasty by TAPP. The histopathological examination showed peritonealized soft tissue with marks of chronic inflammation. Based on the intraoperative and histopathological picture the findings were compatible with a hydrocele of the canal of Nuck. The early postoperative period was uneventful and the patient was discharged home the next day in good clinical condition. On follow up an inguinal hemato-seroma measuring 7 × 4 cm occurred 8 days postoperatively which was self-absorbed. No other complications occurred during a 12-month follow-up.

### 3.4. Case 4

A 35-year-old patient presented in February 2018 with a right-sided inguinal mass that first appeared two years prior. The protruding mass was causing a very unpleasant feeling of pressure to the patient. An MRI was performed at an external radiological institute showed a liquid mass at inguinal area. The most probable diagnosis to that point was a lymphocele. Then the patient was referred to our clinic for further examination. After interdisciplinary discussion of the external MR imaging by radiologists and surgeons of our institution, a type 1 cyst in the canal of Nuck was diagnosed [6].

The patient decided to proceed with elective surgical therapy. The cyst was excised and a right-sided Lichtenstein hernioplasty was performed to cover the hernia defect. The histopathological examination revealed a peritoneal inclusion cyst matching a cyst in the canal of Nuck. The patient was discharged home in stable condition two days after surgery. The patient was asymptomatic in our 6-month follow-up routine.

### 3.5. Case 5

A 41-year-old female was referred to our emergency department in April 2018 for a suspected incarcerated hernia. She complained about pain in the inguinal area. There was no fever, no vomiting, no bowel or bladder dysfunction. Her blood count levels and urinalysis were normal. An irreducible mass containing anechoic fluid was found by ultrasound.

We proceeded with emergency surgical therapy. Laparoscopy revealed an hourglass-shaped Type 3 hydrocele inside the canal of Nuck. A TAPP hernia repair was performed due to widening of the ingunal channel by the hydrocele. The early postoperative period was uneventful and the patient was discharged home two days after surgery in satisfactory condition. The patient remains asymptomatic in our routine follow-up lasting 6 months.

### 3.6. Case 6

**Figure 3:**
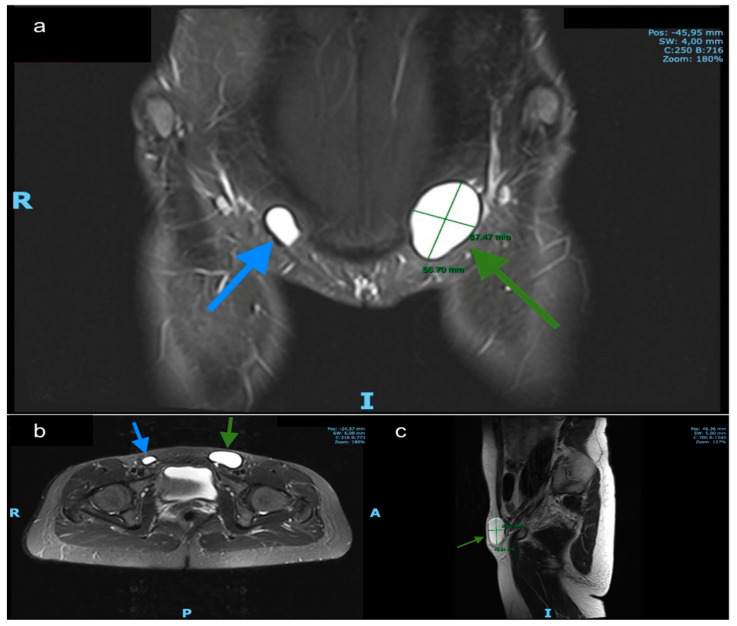
MRI findings. (**a**) Coronal T2-weighted. (**b**) axial T2-weighted and (**c**) sagittal MR images demonstrate a left inguinal hyperintense cystic structure without septation and a smaller (asymptomatic) on the right side. Green arrows: left hydrocele, blue arrows: right hydrocele.

A 34-year-old patient with recurrent groin pain since 2012. Several outpatient consultations passed without a clear diagnosis. She the presented herself at gynecological outpatient department of our hospital in January 2020. In the MRI (T1/T2-weighted with contrast agent) showed a hypointense and hyperintense cystic structure, respectively, without septation of the left groin of 5.6 × 3.7 cm size. A clinically inapparent cyst on the contralateral side was also found (Figure 3). The puncture of the unclear symptomatic cyst revealed histpathologically undefined cell formations.

The patient then presented herself at our surgical outpatient clinic for further examination. We re-evaluated the case and diagnosed a Nuck’s hydrocele on both sides. Intraoperatively, a left-sided Type 2 hydrocele was seen, which could be confirmed by the histopathological finding of a mesothelial covered cystic lesion. The early postoperative period was uneventful and the patient was discharged home the day after surgery in satisfactory condition. Thus far, the patient remains asymptomatic in our routine follow-up. Surgical treatment of the asymptomatic right-sided cyst will also be performed in the course of time in case of discomfort.

**Figure 4:**
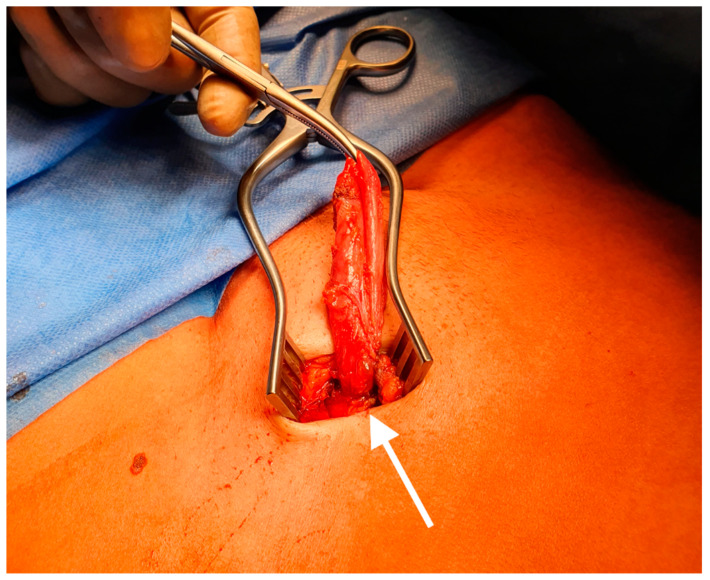
Intraoperative picture of a hydrocele of the canal of Nuck. The round ligament (white arrow).

The demonstrated image material in Figure 3 and Figure 4 originates from this case.

A chart of the cases is shown in Table 1.

### 3.7. Review of the Literature

The selected studies included ten case reports and two retrospective case series. Overall, 39 cases of Nuck Hydroceles have been described in those publications. All patients presented with inguinal swelling. Two patients underwent emergency surgery (5%). The diagnosis was made by Ultrasound (33 patients—85%), Ultrasound and MRI (3 patients—8%), one case with CT and MRI (2.6%) and one case only by clinical examination (2.6%). Of all patients, 26 (67%) underwent laparoscopic hydrocelectomy and high ligation, one patient was treated by laparoscopic TAPP repair (2.6%) and another with laparoscopic total extraperitoneal (TEP) repair (2.6%). Nine patients underwent conventional surgery (23%) and a mesh was implanted in a further three patients (8%). Four patients showed a hernia (10%)—a subdivision in two groups (children and adults) resulted in no case of hernia defects in children but four adults (67% in the adult group). All patients had an uneventful follow-up and showed no recurrence.

An overview of the articles that were included in this review of the literature is shown in Table 2.

## 4. Discussion

The hydrocele of the canal of Nuck in the context of an inguinal swelling is a rare finding and as such it has to be named correctly in terms of terminology after a reliable diagnosis is made. Publications from the nineteenth century show that such cases were often misinterpreted and thus diagnosed and treated as an ordinary inguinal or femoral hernia [18]. For many years, this entity was only presented in individual case reports. The very few case series initially dealt only with the diagnosis and not with the surgical therapy [6,19]. The entity of Nuck’s hydrocele in adults has been covered only by single case reports. No case series regarding adult females with this inguinal pathology has been published.

A variety of masses can be found in the female inguinal region. In summary, the differential diagnosis includes hernia, lymphadenopathy, abscess, Bartholin’s cyst, neurofibroma, sarcoma, liposarcoma, Burkitt lymphoma and posttraumatic/postoperative hematoma [20]. Patients with endometriosis in the canal of Nuck have also been reported [21,22]. Similar findings are conceivable for the canal of Nuck, with single hydroceles or in combination with hernias being the most common one [23].

Furthermore, it is reported that pathologies in the canal of Nuck could be more frequent than previously assumed and should play a more important role in the differential diagnosis of groin pain [1]. The most prevalent conclusion of the studies we reviewed was that Nuck’s should be included in the differential diagnosis of inguinal swelling.

Symptoms can be acute or chronic and infections of the hydroceles are also possible [12,13,14]. Although the majority of published cases did not require emergency surgery, the few cases that did should not be underestimated and left behind [15,24]. Ultrasonography can be the initial imaging because of its low cost and its wide availability and magnetic resonance imaging (MRI) could be used for complex cases and further investigation [1,25,26,27,28]. The sonographic findings mostly show a mass with an elongated morphology, that contains anechoic fluid in the sense of a well-defined unechoic lesion [29]. In the case of a hernia, it may contain omental fat, ovary, fallopian tube, the uterus, parts of the bowel or the urinary bladder [1,23,30]. To differentiate an encysted hydrocele from a hernia, a Valsalva maneuver could be helpful, because it would sometimes change the appearance of a hernia but leave an encysted hydrocele unimpaired [28,31]. As mentioned before, ambiguous sonographic findings can be further investigated with MRI, especially when a herniation is suspected [1]. Our experience with the presented cases showed that the sonographic findings remain undiagnosed if the examiner is not familiar with the possibility of the presence of a Nuck’s hydrocele. The radiological findings have to be confirmed intraoperatively and by the histopathological report.

The hydrocelectomy would be the first surgical step followed by the hernioplasty in case of a hernia. TAPP and Lichtenstein are equivalent and can be used as therapy without any noteworthy limitations [9,10,13]. Most likely, the results of a comparison between the two methods will be similar to those found in the meta-analysis by Scheuermann et al. in inguinal hernia in males, showing that TAPP is associated with less chronic inguinal pain in comparison with Lichtenstein repair [32]. In addition, the TEP approach is an alternative that could also be useful [13]. A comparison between TAPP and TEP will most likely show comparable outcomes for the two techniques with advantages on the TAPP side regarding operation time and conversion rates [33,34,35]. Furthermore, it is an accepted opinion that, when using the TEP technique, it is more difficult to identify anatomic landmarks compared to the TAPP technique and, therefore, this method is not suitable for exploration [34]. Sonographically guided aspiration of the cyst could be used to temporarily alleviate patient discomfort, especially in elderly patients, who probably would not circumvent a surgery [36].

## 5. Conclusions

We can report that all our patients have benefited from the treatment in terms of their symptoms and, so far, we report no recurrence of the hydroceles. The maximum postoperative hospital stay was two days and the follow-up six months postoperatively has been uncomplicated. We are now sensitized by the experiences we have made in our clinic, mostly through random findings, and we believe that our case series could be used as a benchmark for further studies with larger case series and possibly register data. Therefore, although being rather rare, Nuck’s hydroceles should be included in the differential diagnosis of inguinal swelling. Taking the classification into consideration, we recommend a conventional approach for the encysted, external type 1 and a laparoscopic approach for the intra-abdominal type 2 Nuck’s hydroceles. Type 3 has to be evaluated individually as it is challenging and the surgical outcome is depending on the surgeon’s skills. A fascial augmentation with mesh, as performed in inguinal hernia surgery, should be considered if inguinal channel has been widened or fascial fibers are damaged by the presence of a Nuck hydrocele.

## Figures and Tables

**Table 1:** Chart with patients treated in our institution due to a cyst of the canal of Nuck.

	Patient 1	Patient 2	Patient 3	Patient 4	Patient 5	Patient 6
Age (years)	29	29	44	35	41	34
Symptoms/Reason of Presentation	Inguinal pain	Asymptomatic inguinal swelling	Inguinal pain and swelling	Inguinal pain and swelling	Inguinal pain and swelling	Inguinal pain and swelling
Diagnostic Study	Ultrasound	Ultrasound	Ultrasound	Ultrasound and magnetic resonance imaging (MRI)	Ultrasound	MRI
Elective/Emergent	Elective	Elective	Elective	Elective	Emergent	Elective
Procedure	Hydrocelectomy and TAPP hernioplasty	Hydrocelectomy and TAPP hernioplasty	Hydrocelectomy and TAPP hernioplasty	Hydrocelectomy and Lichtenstein hernia repair	Hydrocelectomy and TAPP hernioplasty	Open hydrocelectomy and fascial suture
Operating Time (min)	52 min	62 min	62 min	49 min	65 min	27 min
Type of Nuck hydrocele	type 2	type 2	type 2	type 1	type 3	type 2
Postoperative-Hospital Stay (days)	2	2	2	2	2	1
Complication	inguinal seroma	none	inguinal hemato-seroma	none	none	none
Recurrence	no	no	no	no	no	no
Follow up Period (months)	1, 3 and 6	1, 4 and 6	6	6	3 and 6	1 and 6

**Table 2:** Comparison of the literature referring to Nuck hydroceles with surgical treatment.

	Year of Publication	Children/Adult	Type of Publication	Diagnostic Study	Elective/Emergent	Procedure	Presence of Hernia	Hospital Stay	Follow-Up	Author’s Conclusion
Kim et al. [8]	2016	adult	Single case report	CT and magnetic resonance imaging (MRI)	elective	Open Hydrocelectomy with high ligation	unknown	unknown	unknown	Inclusion in differential diagnosis of inguinal swelling.
Janssen et al. [4]	2011	child	Single case report	none	elective	Open Hydrocelectomy with high ligation	unknown	unknown	uneventful, no recurrence	Inclusion in differential diagnosis of inguinal swelling.
Qureshi et al. [9]	2013	adult	Single case report	Ultrasound	elective	Laparoscopic hydrocelectomy and hernia repair with mesh	yes	unknown	uneventful, no recurrence	-
Lombardo et al. [10]	2017	adult	Single case report	Ultrasound and MRI	elective	Open hydrocelectomy and hernia repair with mesh	yes	unknown	uneventful, no recurrence	-
Topal et al. [11]	2018	adult	Single case report	Ultrasound and MRI	elective	Open hydrocelectomy and hernia repair with mesh	yes	1 day	unknown	Inclusion in differential diagnosis of inguinal swelling.
Mandhan et al. [12]	2013	child	Single case report	Ultrasound	emergent	Open Hydrocelectomy with high ligation	no	unknown	uneventful, no recurrence	Inclusion in differential diagnosis of inguinal swelling. Surgery is mandatory for final diagnosis an treatment.
Matsumoto et al. [13]	2014	adult	Single case report	Ultrasound and MRI	elective	Hydrocelectomy and hernia repair with mesh (TEP)	yes	3 days	unknown	TEP approach with its advantage of a shorter recovery period could be useful
Sarkar et al. [14]	2014	child	Single case report	Ultrasound	emergent	Open Hydrocelectomy with high ligation	no	unknown	uneventful, no recurrence	Inclusion in differential diagnosis for inguinal swelling. U/S and MRI preoperatively. Surgery is the treatment of choice.
Lucas et al. [15]	2019	adult	Single case report	Ultrasound	elective	Open Hydrocelectomy with high ligation	no	unknown	uneventful, no recurrence	Inclusion in differential diagnosis of inguinal swelling.
Lee et al. [7]	2018	children	Case series (26 patients)	not enough information	not enough information	Laparoscopic hydrocelectomy with high ligation	none	on average 7.5 h	uneventful, no recurrence	Lap. Hydrocelectomy with high ligation is an effective method for treating Nuck´s hydroceles in pediatric patients
Caviezel et al. [16]	2009	adult	Case report	MRI	elective	Open Hydrocelectomy	no	unknown	uneventful, no recurrence	Inclusion in differential diagnosis of inguinal swelling, Ultrasound for a first evaluation and MRI for precise information
Papparella et al. [17]	2018	Children	Case series (353 patients, of whom 3 had a Nuck hydrocele)	Ultrasound	elective	Open Hydrocelectomy with high ligation	no	unknown	uneventful, no recurrence	Surgery with high ligation is considered as therapy of choice for Nuck’s hydroceles

## Supplementary Materials

The following are available online at https://www.mdpi.com/2077-0383/9/12/4026/s1, Video S1: TAPP repair of Nuck´s hydrocele
